# Mild and repetitive very mild axonal stretch injury triggers cystoskeletal mislocalization and growth cone collapse

**DOI:** 10.1371/journal.pone.0176997

**Published:** 2017-05-04

**Authors:** Yiing C. Yap, Anna E. King, Rosanne M. Guijt, Tongcui Jiang, Catherine A. Blizzard, Michael C. Breadmore, Tracey C. Dickson

**Affiliations:** 1 Menzies Institute for Medical Research, University of Tasmania, Tasmania, Australia; 2 Wicking Dementia Research and Education Centre, University of Tasmania, Tasmania, Australia; 3 Pharmacy School of Medicine, Australian Centre for Research on Separation Science (ACROSS), University of Tasmania, Tasmania, Australia; 4 ACROSS, School of Physical Sciences, University of Tasmania, Tasmania, Australia; Michigan State University, UNITED STATES

## Abstract

Diffuse axonal injury is a hallmark pathological consequence of non-penetrative traumatic brain injury (TBI) and yet the axonal responses to stretch injury are not fully understood at the cellular level. Here, we investigated the effects of mild (5%), very mild (0.5%) and repetitive very mild (2×0.5%) axonal stretch injury on primary cortical neurons using a recently developed compartmentalized *in vitro* model. We found that very mild and mild levels of stretch injury resulted in the formation of smaller growth cones at the tips of axons and a significantly higher number of collapsed structures compared to those present in uninjured cultures, when measured at both 24 h and 72 h post injury. Immunocytochemistry studies revealed that at 72 h following mild injury the axonal growth cones had a significantly higher colocalization of *β*III tubulin and F-actin and higher percentage of collapsed morphology than those present following a very mild injury. Interestingly, cultures that received a second very mild stretch injury, 24 h after the first insult, had a further increased proportion of growth cone collapse and increased *β*III tubulin and F-actin colocalization, compared with a single very mild injury at 72 h PI. In addition, our results demonstrated that microtubule stabilization of axons using brain penetrant Epothilone D (EpoD) (100 nM) resulted in a significant reduction in the number of fragmented axons following mild injury. Collectively, these results suggest that mild and very mild stretch injury to a very localized region of the cortical axon is able to trigger a degenerative response characterized by growth cone collapse and significant abnormal cytoskeletal rearrangement. Furthermore, repetitive very mild stretch injury significantly exacerbated this response. Results suggest that axonal degeneration following stretch injury involves destabilization of the microtubule cytoskeleton and hence treatment with EpoD reduced fragmentation. Together, these results contribute a better understanding of the pathogenesis of mild and repetitive TBI and highlight the therapeutic effect of microtubule targeted drugs on distal part of neurons using a compartmentalized culturing model.

## Introduction

Diffuse axonal injury (DAI) throughout the white matter is a common and important feature of traumatic brain injury (TBI) [1]. It is thought to be caused by rapid brain deformation, compression or stretching as a result of traumatic incidents rather than a penetrative injury that causes complete neuronal transection and disconnection. Over the past few years, public awareness of the consequences of mild traumatic brain injury (mTBI) and concussion has increased. Determining how the axon responds to such mild, and/or repetitive insults could reveal important opportunities for therapeutic interventions targeted at halting pathological cascades and preserving neuronal function. Essential to this goal is a detailed understanding of the axonal response to defined insults. To this end, in order to identify the abnormal axonal alterations in response to repetitive mTBI, researchers have developed animal models such as the controlled cortical impact [2, 3] and weight drop [4] to experimentally induce axonal stretch or compression injuries. These models have demonstrated exacerbated outcomes of impaired cognitive function and axonal injury with repetitive mTBI compared to a single mTBI [2–4]. For example, Huh and colleagues [2] demonstrated that repetitive mild, non-contusive head injury, using a controlled cortical impact model in 11-day old rats, resulted in axonal disconnection at 3 days following single impact, while double and triple impacts produced axonal disconnections at 1 day post-injury. While animal models have yielded considerable insight regarding the changes in animal behaviour and axonal alterations in response to repetitive mTBI, their limitation is that they only postulate the underlying mechanisms of cognitive impairment at the cellular level. In addition, these animal models cannot distinguish if a worse outcome after repetitive injury is simply due to a cumulative effect or reflects a mechanism of exacerbated outcome following first injury.

A number of *in vitro* models of axonal stretch injury have been developed and utilized in order to facilitate the investigation of various pathobiological mechanisms at both the cellular and subcellular levels. For example, Ellis *et al*. [5] developed a stretch injury model and defined a mild injury as a 5.5 mm deformation (or 31% membrane strain) of the flexible silicone membrane following the application of a constant air pressure pulse. Slemmer and colleagues [6] used this model to examine the cellular events following repetitive mTBI. They showed that repeated mild injury resulted in significantly increased apoptosis as compared with a single injury, suggesting cumulative damage to the brain following multiple mild injuries. However, this method cannot be used to study the spatial compartmentalization signals due to the random spatial distribution of neurons on the silicon membrane. Furthermore, the testing of therapeutic strategies that aid in axon protection or regeneration following injury is also limited due to the difficulty in isolating axons. More recently, Smith and co-workers [7] developed another *in vitro* model system that induces dynamic stretch of isolated axons spanning two populations of neurons. A deformable silicon membrane was placed on the stainless steel plate with a machined 2 × 18 mm slit. The slit in the plate is then aligned with the axon only region on the silicon membrane, at the bottom of an airtight chamber. A controlled air pulse was used to rapidly change the chamber pressure and deflect the portion of the silicon membrane that contains the cultured axons downward, inducing tensile elongation. Subsequently, Yuen *et al*. [8] utilized this model to examine the effect of repetitive mild axonal stretch injury. When they applied a single, mild strain (3%) at rates of 20 s^-1^ to rat cortical axons in culture, no obvious pathological change was observed, however, the axons were found to display increased sodium channel expression by 24 h. When they applied a second, identical mild injury 24 h after the first injury, a significant increase in intracellular calcium was observed, which then lead to axon degeneration. These findings suggest that initial mTBI triggers a pathophysiological response that makes neurons more susceptible to an exaggerated outcome from a subsequent mTBI. However, this model only allows the physical isolation of axons from soma, but does not offer the ability to control different neuronal compartment microenvironments.

Here, we have utilized our previously developed novel *in vitro* model of axonal stretch injury [9] to investigate axonal responses to single stretch injury and repetitive injury in a fluidically isolated microenvironment. The microfluidic device physically isolates axons from the soma as well as fluidically controls the axon and soma compartments’ microenvironments using narrow microgrooves (10 *μ*m wide, 3 *μ*m high, 450 *μ*m wide) [10]. Furthermore, it has been modified by integrating a pneumatic channel [11] which allows application of a standardised, precise and highly localised injury to the axon [9]. A thin poly (dimethylsiloxane) (PDMS) membrane was irreversibly bonded with the pneumatic channel device and placed underneath the microfluidic culturing device. When the pneumatic channel (90 *μ*m wide, 17 *μ*m high) is pressurized, the flexible thin PDMS membrane deflects upward, stretching the axons growing on top. Using this platform, very mild (0.5% strain) and mild (5% strain) stretch injuries can be applied to a 90 *μ*m long section of the axons [9]. We compared the morphology and cytoskeletal profile of growth cones on the tips of the axons following mild (5%), very mild (0.5%), and repetitive very mild (2×0.5%) axonal stretch injury using this platform. We also exploited the fluid isolation afforded by this platform to investigate the role of microtubules in these alterations and identified the potential use of microtubule stabilizing agent-Epothilone D (EpoD) for the protection of axons from stretch injury-induced degenerative response.

## Materials and methods

### Stretch injury microfluidic device

**Fig 1:**
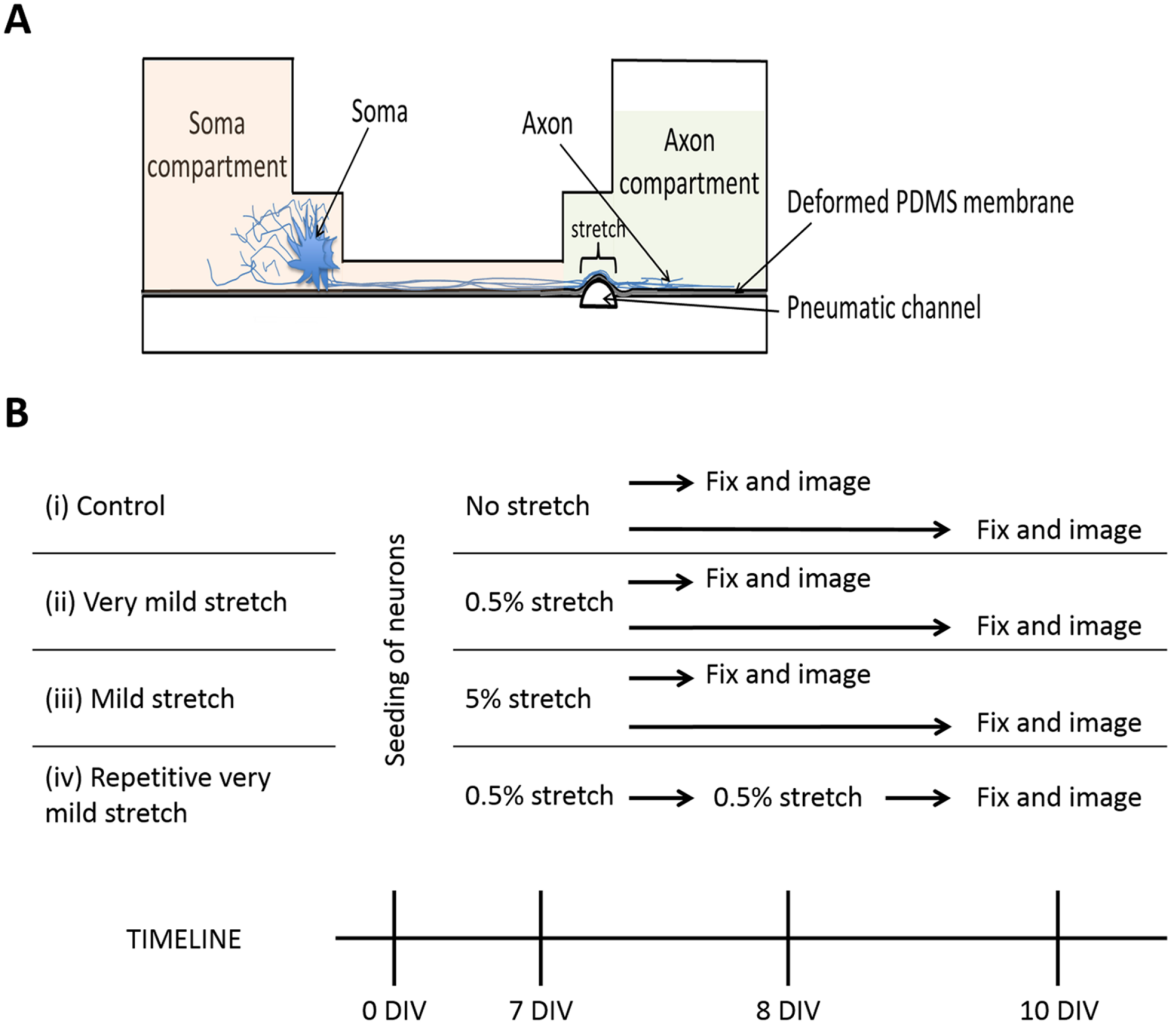
**(A) Stretch injury microfluidic device.** Cell bodies or soma are isolated in the soma compartment and axons extend into the axon compartment through microgrooves (10 *μ*m width and 3 *μ*m high). A gas pressure is applied into the pneumatic channel and deforms the thin PDMS membrane, causing the stretching of axons growing on top. **(B) Timeline for the control, very mild (0.5%), mild (5%) and repetitive very mild (2×0.5%) stretch injury experiments.** (i) For the control, neurons were seeded and grew on the stretch injury microfluidic device without applying any gas pressure, and fixed and imaged at both DIV 8 and DIV 10. (ii), (iii) Isolated axons were stretched at 0.5% strain or 5% strain at DIV 7 and fixed and imaged at both DIV 8 and DIV 10. (iv) For the repetitive injury investigation, isolated axons initially received 0.5% stretch on DIV 7. 24 h later, a second 0.5% stretch injury was applied on the same device and then fixed at DIV 10.

A novel *in vitro* model was used to induce a very mild (0.5%) to mild (5%) axon injury in primary cultured neurons [9]. This device consists of two independent PDMS structures separated by a 60 ***μ***m or 15 ***μ***m thick PDMS membrane (Fig 1A). Rat cortical neurons are grown in the upper PDMS microfluidic culturing device (Xona Microfluidic, CA), which has two microfluidic compartments of 100 ***μ***m height, 1.5 mm width and 8 mm length interconnected with microgrooves of 10 ***μ***m width, 3 ***μ***m height and 450 ***μ***m length. The small size of the microgrooves prevents migration of cell bodies between the compartments while allowing only axons to pass through [12]. The bottom structure contains a pneumatic channel (17 ***μ***m high, 90 ***μ***m wide, 40 mm long) and is irreversibly sealed with the PDMS membrane using a handheld corona discharge unit (Electro Technic Product Inc, USA). The pneumatic valve microfluidic device was replicated in PDMS (Sylgard 184, Dow corning, Michigan, USA) by soft lithography and replica molding procedure from a patterned lithographic dry film master. In response to a controlled pressure pulse, the pneumatic channel inflates and the PDMS membrane deflects upward, stretching the axons growing on top to varying degrees.

### Preparation of stretch injury microfluidic device prior to culturing

Stretch injury microfluidic devices (Fig 1A) were prepared as described previously [9]. Briefly devices were sterilized with 70% ethanol and ultra-violet (UV) light. The surface of the PDMS pneumatic channel device was first hydrophilized using a handheld airplasma unit and then attached to the compartmented culturing microfluidic device. Poly-L-lysine (PLL) (0.001%, Sigma, USA) was loaded into both compartments of the culturing microfluidic device and incubated for at least 3 days at room temperature to allow adequate coating. PLL was removed and culturing devices were filled with initial neuronal growth media consisting of Neurobasal^™^, 10% heat inactivated foetal calf serum, 2% B27 supplement, 0.5 mM glutamax, 25 *μ*m glutamate and 1% penicillin-streptomycin (Gibco/BRL, Life Technologies, USA). The culturing devices were equilibrated in a cell culture incubator (37°C, 5% CO_2_) for a minimum of 24 h prior to the addition of cells.

### Primary cortical neuron culture

All animal experimentation was approved by the Animal Ethics Committee of the University of Tasmania and is consistent with the Australian Code of Practice for the Care and Use of Animals for Scientific Purposes. Sprague Dawley rats were initially sourced from Monash University, and are maintained as an outbred colony with breeding males replaced every five generations. Rats are housed in microisolator cages on a 12-h light/dark cycle with free access to food and water and are euthanized with CO_2_ (infusion rate 7.3 liters per minute, for 5 minutes). Cortical neurons were prepared from the cerebral cortices of embryonic day 18 (E18) Sprague Dawley rat embryos as previously reported [9]. Briefly the dissected cells were trypsinised (0.0125%) followed by washing and gentle physical dissociation with a 1 ml pipette. Cell viability and density was assessed by trypan blue exclusion assays. Media was removed from the wells of the microfluidic devices and cells (10 *μ*l) were then loaded into the soma compartment of the devices at a density of 8×10^6^ cells/device. Devices with cells were then placed in a humified incubator at 37°C, 5% CO_2_ for 5 min to enhance cell adhesion to the PDMS substrate. After 5 min, both soma and axon compartments were filled slowly with pre-warmed initial neuronal growth media and returned to the incubator. After 24 h, the media was replaced with subsequent growth media (initial growth media without the foetal calf serum and glutamate). The cell culture was then monitored at regular intervals and the media replaced every second day to prevent oxygen and nutrient depletion and/or waste accumulation.

### Axonal stretch injury

Stretch injury was applied to a localised region within the axon compartment at 7 days following plating of primary cortical neurons in the microfluidic device by applying gas pressure to the pneumatic channel underneath the flexible PDMS membrane. Measurement of the percentage stretch applied was calculated as the percentage increase in length of membrane following stretch compared to the original length of membrane. The half-length of membrane following stretch (*L*) is determined based on the half-length of the original length of the membrane (*w*) and deflected height (*h*) using Phythagoras Theorem where *w*^2^ + *h*^2^ = *L*^2^. Two levels of stretch injury were performed (4.3 and 14.1 *μ*m deformations) on 90 *μ*m long membrane and defined as “very mild” and “mild” respectively. These deformations resulted in a stretch of 0.5% and 5% respectively. Axons received mild stretch (5%) or very mild stretch (0.5%) at 7 DIV and were then fixed and imaged at both 8 DIV and 10 DIV. For repetitive injury investigations, cultures received the double, very mild injury (2×0.5%) stretch injury on day 7 and then again 24 h after the first stretch injury event. Cultures were then fixed and imaged at 10 DIV. Sham-injury or control cultures were grown on the stretch injury device without applying the gas pressure and fixed and imaged at 8 DIV and 10 DIV (Fig 1B).

### Pharmacological manipulation

For the investigation of targeted EpoD treatment, The 0.1 nM, 1 nM, 10 nM or 100 nM EpoD or dimethyl sulfoxide (DMSO; Sigma) vehicle control alone was added to the axon compartment immediately following mild axonal stretch injury (5%) at 7 DIV. Cultures were then fixed and imaged after 24 h following drug or vehicle treatment.

### Immunocytochemistry

Following injury or drug treatment, cells were fixed with 4% paraformaldehyde (PFA) in phosphate buffered saline (PBS) for 30 min at room temperature and then permeabilized with 0.3% Triton X-100 for 15 min. This was followed by incubation with primary antibodies diluted in PBS for 1 hour at room temperature and then overnight at 4°C. Primary antibodies included *β*III-tubulin (1: 1000, mouse monoclonal, G7121, Promega, USA) and microtubule associated protein Tau (1: 5000, rabbit polyclonal, A0024, Dako, Denmark). Secondary antibodies (Mouse IgG AlexaFluor 488, A11029 and Rabbit IgG AlexaFluor 488, A11034, 1: 1000, ThermoFisher Scientific, USA) were applied for 2 h at room temperature in the dark. To label filamentous actin (F-actin), cultures were incubated with AlexaFluor 594 phalloidin (1: 200, A12379, ThermoFisher Scientific, USA) for 30 min in dark after primary and secondary antibodies labelling.

### Quantitative analysis

Fixed, fluorescently labelled samples were visualized with a Leica DMLB2 fluorescent microscope (Leika, Germany) and images were acquired with a CCD camera (ORCA, Japan) and recorded in NIH elements software (Nikon, Japan). To quantitatively examine the organization of microtubules and actin filaments within the growth cones of uninjured and stretch injured neurons, 100X images of *β*III tubulin labelling were merged with images of phalloidin staining. Growth cones were classified as either “not collapsed” (with filopodia extension and lamellipodia) or “collapsed” (without lamellipodia and with ≤3 retraction fibers) [13]. The total percentage of growth cone collapse was calculated as (number of collapsed growth cones/total number growth cones) × 100. The average area of growth cones in both uninjured and stretch-injured cultures was measured by framing the actin positive extension using the freehand modus of NIH Image J software as described previously by Richter and colleagues [13] (S1 Fig). The amount of colocalization between microtubules and F actin was determined using the Image J-JacoP colocalization plug-in function.

To quantify axonal degeneration following injury, with and without treatment with EpoD, we used the method described by Sasaki and colleagues [14]. Here, we analysed axonal degeneration by comparing axonal tau labelling among experimental groups. Tau is a microtubule associated protein localised specifically to axons and routinely used to visualise these processes [15]. Briefly, each tau labelled fluorescent image (40X) was analysed by NIH imageJ software. To obtain the total axon area, images were binarized. The total axonal area was determined by the total number of detected black pixels after the image was binarized. The area of degenerated axon fragments was calculated using particle analyser algorithm of ImageJ with circularity more than 0.2 determined and designated as fragmented [14]. A degenerative index (DI) was calculated as the ratio of fragmented axon area over total axon area.

Three randomly selected fields from the distal axon compartment of the same microfluidic device were imaged for quantification and n = 3 devices from three separate cultures were used for all analysis. The results are presented as the mean ± standard error of mean (SEM). All statistical tests were made using one way ANOVA with post hoc Fisher’s LSD test, with p values less than 0.05 as the level of significance.

## Results

### Compartmentalized culture of axons and cell bodies in a stretch injury microfluidic device

**Fig 2:**
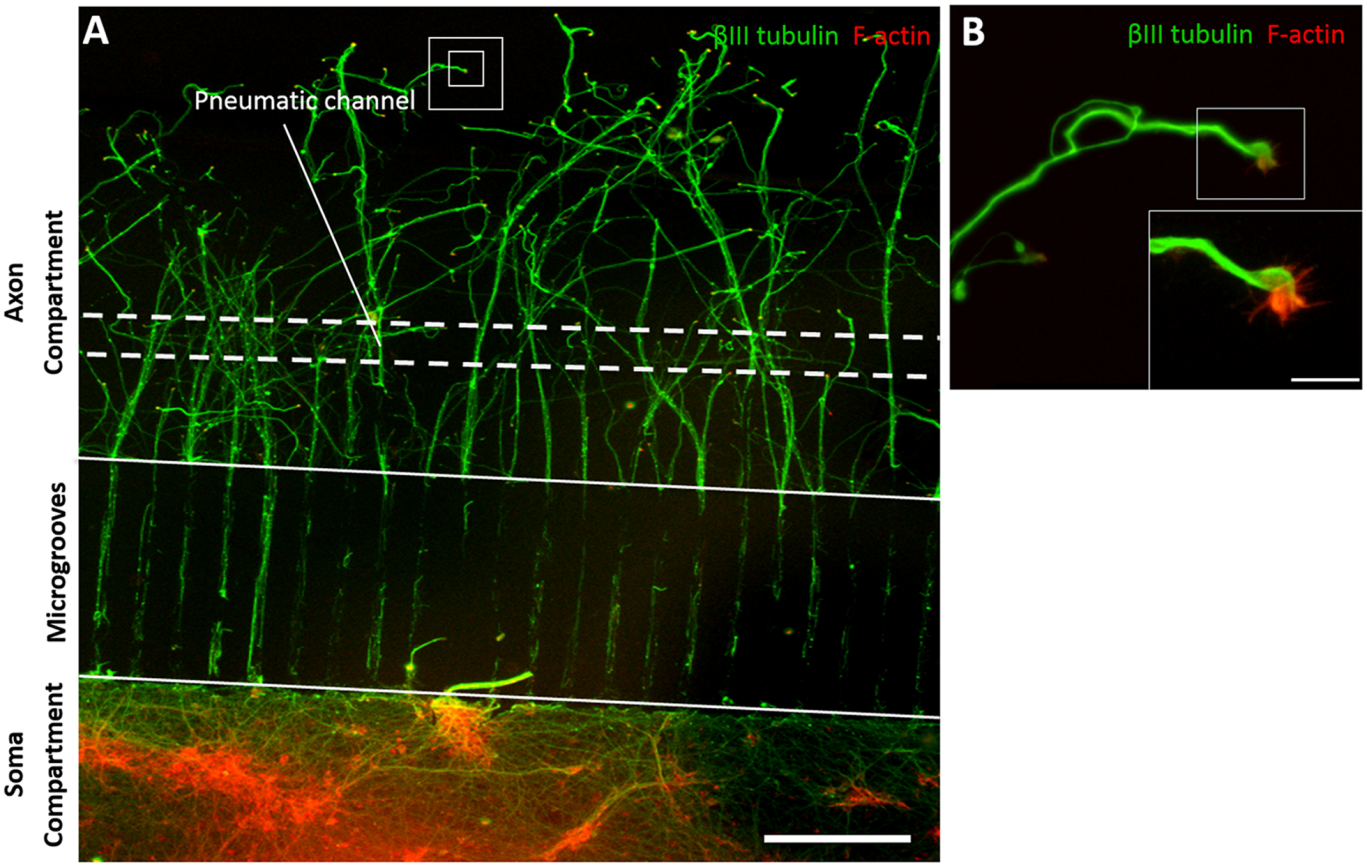
Double immunolabelling verified extensive network of axons (*β*III tubulin immunoreactivity) and growth cones (F-actin staining) within the axon compartment of the stretch injury model at 7 DIV. **(A)** Axons (green; *β*III tubulin) of primary rat cortical neurons extended into the axon compartment through microgrooves (450 *μ*m long, 10 *μ*m width and 3 *μ*m high) at 7 DIV. Dashed lines indicate the location of the pneumatic channel (or stretch injury) and solid lines show the microgrooves region. **(B)** High magnification of axons with growth cones immunostained with F-actin (red) and microtubules (green) (white square box in panel A). Insets show higher magnification of growth cone. Scale Bars = 200 *μ*m (A), 75 *μ*m (B), 40 *μ*m (insets).

To further define the sequence of pathological changes that characterise the axonal response to injury, we used an *in vitro* microfluidic device of isolated axonal stretch injury to simulate mild (5%) and very mild (0.5%) stretch injury of axons by incorporating microfluidic valve technology into a compartmented microfluidic culturing device [9]. This stretch injury microfluidic device was combined with a previously established two compartments microfluidic culturing device designed by Taylor and coworkers [12], allowing axon outgrowth and compartmentalization of the culture. We demonstrated that cell bodies were restricted to the soma compartment, and only axons began to extend into the axon compartment at 7 DIV. Double immunolabeling verified an extensive network of axons (*β*III tubulin immunoreactivity) and growth cones (F-actin staining) within the axon compartment of the stretch injury microfluidic device at 7 DIV (Fig 2A and 2B), allowing us to study the response of the tip of the axons in the distal axon compartment following localized, distal axonal stretch injury through the pneumatic channel. In addition, the embedded microgrooves that separated soma compartment and axon compartment in the compartmented microfluidic device allowed growth of the axons into a fluidically isolated microenvironment. Therefore, pharmacological treatment such as Epo D can be performed in a highly targeted manner, to the axons and/or the soma.

### Stretch injured axons formed smaller growth cones

**Fig 3:**
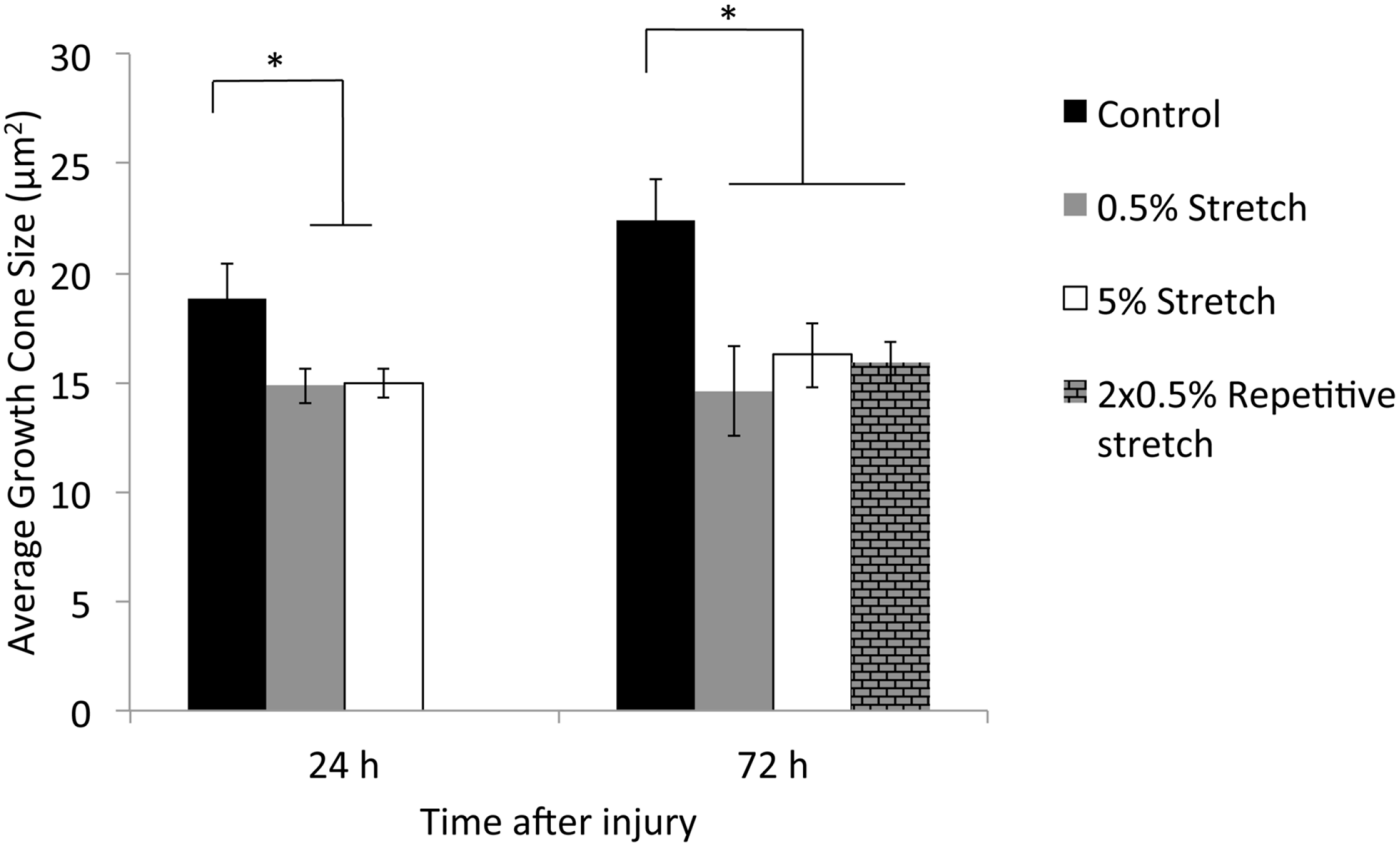
Graphs showing average growth cone area of control, 0.5% stretched, 5% stretched and repetitive very mild (2×0.5%) stretched axons at different time point. There was a significant decrease in growth cone size after single stretch injury at both 24 h and 72 h PI and after repetitive very mild (2×0.5%) stretch injury at 72 h PI compared to control. *p<0.05. Error bar = mean ± SEM.

Examination of cultures that received stretch injury revealed a distinct difference in the size of the growth cones on the tips of the axons in the distal axon compartment, compared to uninjured neurons. To quantitatively investigate this observation we determined the size of the growth cones in uninjured and stretched injured cultures by measuring their area, using the F-actin stain, phalloidin. Our results showed that the area of the growth cone following 0.5% stretch injury (14.98 ± 0.78 *μ*m^2^) and 5% stretch injury (14.86 ± 0.65 *μ*m^2^) at 24 h PI was significantly smaller compared to the area of the growth cones in the control, uninjured neurons (18.85 ± 1.53 *μ*m^2^) (p<0.05, Fig 3). However, there was no significant difference between the size of the growth cones following 0.5% and 5% stretch injury at 24 h PI. Similarly, the area of the growth cones following 0.5% stretch injury (14.59 ± 2.03 *μ*m^2^) and 5% injury (16.25 ± 1.49) *μ*m^2^) was significantly smaller than the area of growth cones in control, uninjured neurons (22.40 ± 1.89 *μ*m^2^) at 72 h PI (p<0.05, Fig 3). There was also no significant difference between the size of growth cones following 0.5% and 5% injury at 72 h PI. Taken together, these results indicate that growth cones in cultures following both mild and very mild axonal stretch injury were not significantly different but they were significantly smaller compared to growth cones in uninjured cultures at both times point examined.

### Cytoskeletal profile changes were observed in axonal growth cones following stretch injury

**Fig 4:**
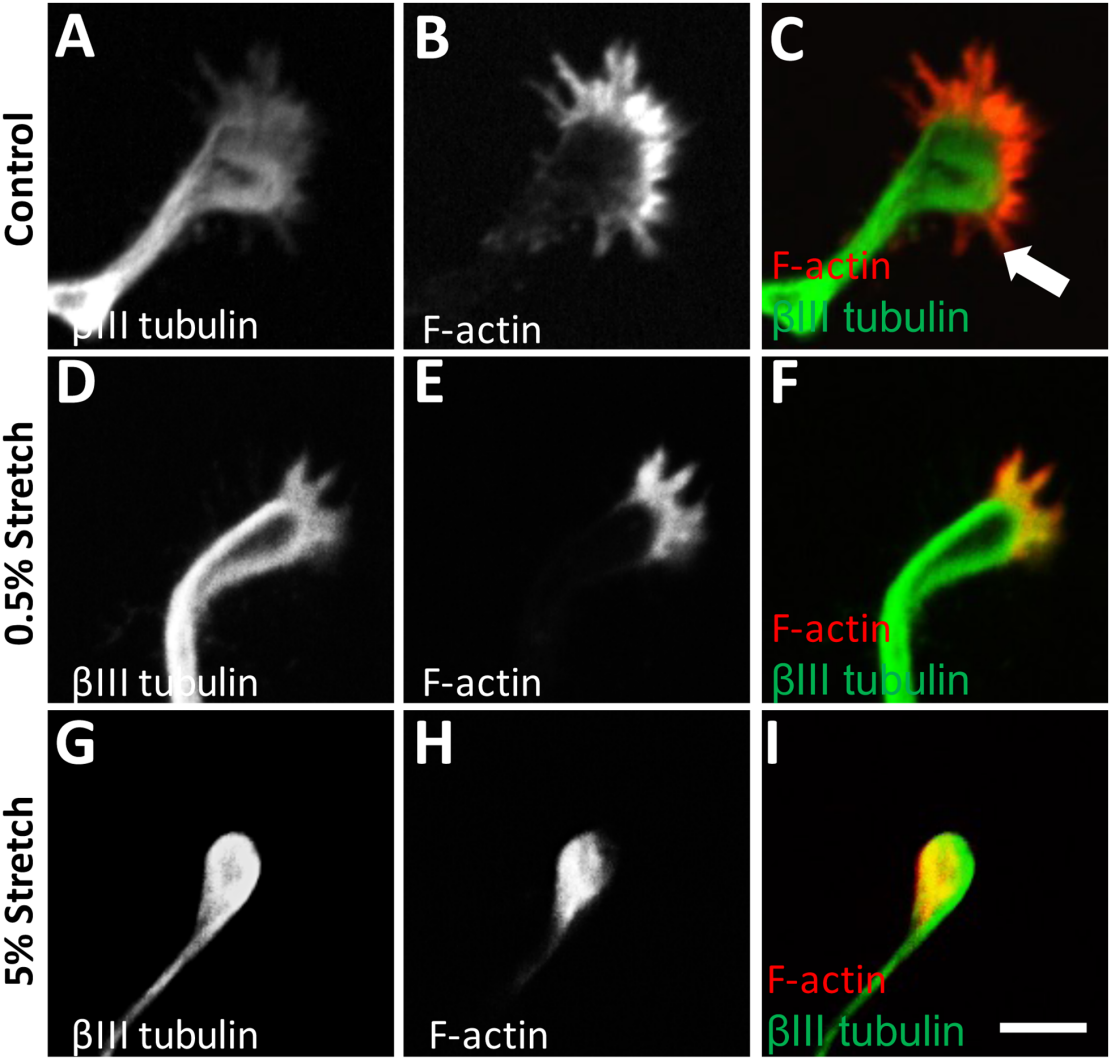
Immunocytochemistry images of growth cones of control (uninjured), 0.5% stretched and 5% stretched axons at 10 DIV and 72 h PI. **(A-C)** In the control, growth cones with distinct filopodia were apparent (arrow). The *β*III tubulin labelling (green) was distributed predominantly within the central domain of the growth cone while F actin (red) was confined to the peripheral region. **(D-F)** In the 0.5% stretched axon, microtubules appeared to form a loop in the central region of the growth cones and F-actin was confined to the peripheral and transition region only. **(G-I)** In the 5% stretched axon, F-actin was most abundant in the axon tip, forming bulb like accumulations. Scale Bars = 10 *μ*m.

We have demonstrated that the size of growth cones following axonal stretch injury was smaller than the growth cones in the uninjured neurons. In order to examine whether these smaller growth cones exhibit different cytoskeletal profiles, we investigated the cytoskeletal changes of the growth cones in the uninjured, control culture and also cultures after axonal stretch injury by examining the distribution of actin and microtubules. The growth cones were labelled with both phalloidin (F-actin stain) and *β*III tubulin (microtubule marker). *β*III tubulin was found to be confined to the central domain, while F-actin was localized to the peripheral domain and the distal tips of filopodia throughout control, uninjured growth cones at 10 DIV (Fig 4A–4C). However, we observed that the central region of microtubules of growth cones at 72 h following very mild stretch injury (0.5%) appeared to form a loop (Fig 4D–4F). At the same time point, 72 h after mild stretch injury (5%), many of the growth cones at the proximal tip of the axon had a collapsed morphology where the growth cones was oval in shape and lacked filopodia extensions (Fig 4G–4I).

### Collapsed growth cones were increased following stretch injury

**Fig 5:**
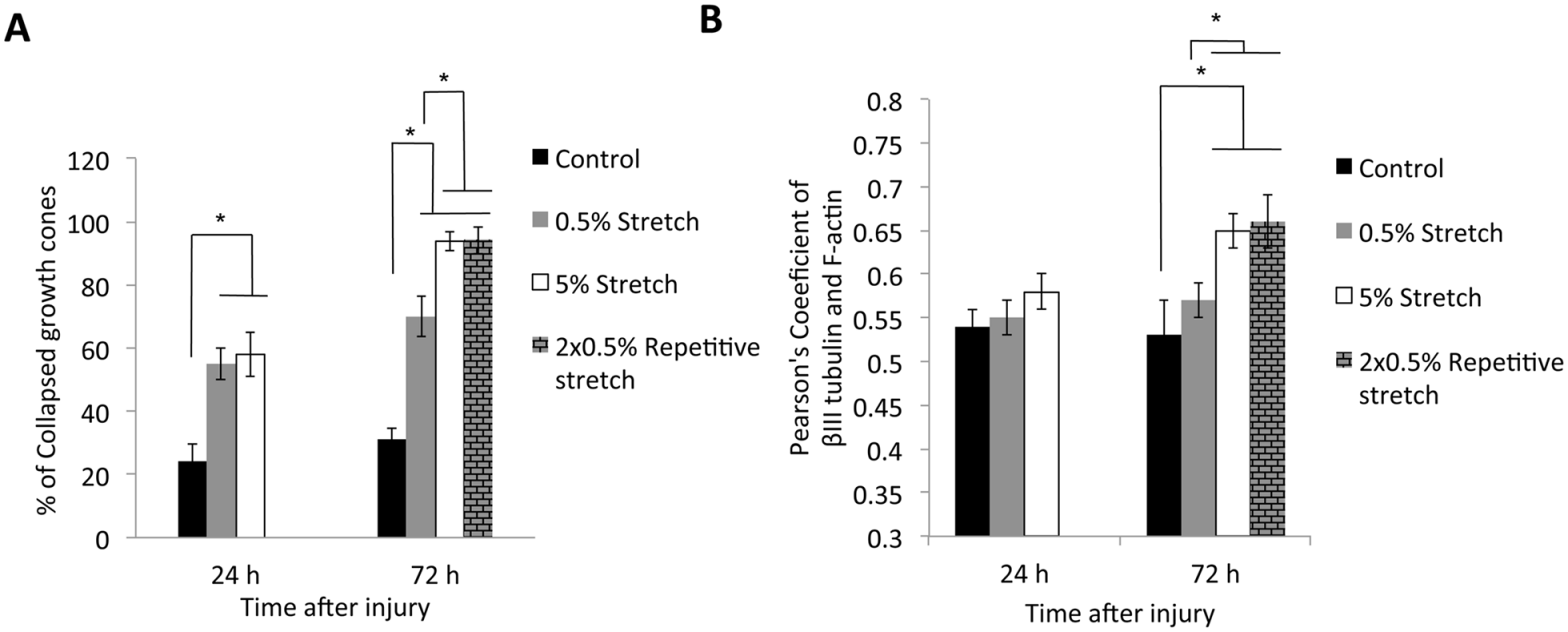
Graphs showing the mean percentages of collapse growth cone and the extent of colocalizatuon of F actin and *β*III tubulin in growth cones of control cultures and cultures after 0.5% stretched, 5% stretched and repetitive very mild (2×0.5%) stretched axons at different time point. **(A)** Stretch injury induced increased axonal growth cone collapsed at both 24 h and 72 h PI compared to the control. In addition, repetitive very mild (2×0.5%) stretch injury induced more collapsed growth cones when compared to single 0.5% stretched axon at 72 h PI. **(B)** The growth cones in 5% stretched axon had significantly higher colocalization value of *β*III tubulin and F-actin compared to both the growth cones in control and 0.5% stretched axon at 72 h PI. However, there was no significant difference between the growth cones in control, 0.5% stretched or 5% stretched axon at 24 h PI. The growth cones in 2×0.5% repetitive stretched axon has significantly higher colocalization value of *β*III tubulin and F-actin if compare to both the growth cones in control and single 0.5% stretched axon at 72 h PI. *p<0.05. Error bar = mean ± SEM.

Trauma such as axotomy is known to cause growth cone collapse [16]. In order to investigate the number of collapsed growth cones following stretch injury, we quantitatively measured the proportion of collapsed growth cones in all conditions. We found that the proportion of collapsed growth cones following 0.5% (50.29 ± 4.98% at 24 h and 68.33 ± 6.31% at 72 h) and 5% injury (64.09 ± 7.01% at 24 h and 93.89 ± 3.09% at 72 h) was significantly higher compared to control cultures (24.09 ± 5.43% at 24 h and 32.38 ± 3.72% at 72 h) at both time points examined (p<0.05, Fig 5A). There was no significant difference between the percentage of collapsed growth cones at 24 h following 0.5% stretch injury and 5% stretch injury. However, the 5% stretch injury resulted in significantly higher proportion of collapsed growth cones compared to 0.5% injury at 72 h PI.

### Distribution of actin and microtubules were significantly altered in growth cones after axonal stretch injury

To further examine the changes in microtubule and actin organization following stretch injury, we quantitatively measured the extent of colocalization of F-actin and *β*III tubulin in growth cones of control cultures and in cultures after mild (5%) and very mild (0.5%) axonal stretch injury. At 24 h PI, the colocalization of F-actin and *β*III tubulin throughout the growth cones of axons following 0.5% (Pearson’s coefficient = 0.55 ± 0.02) or 5% (Pearson’s coefficient = 0.58 ± 0.02) stretch injury was not significantly different to the control, uninjured axons (Pearson’s coefficient = 0.54 ± 0.02). In addition, we also observed that colocalization of *β*III tubulin and F-actin in growth cone following 0.5% stretch injury (Pearson’s coefficient = 0.57 ± 0.03) was similar to the growth cones in control culture (Pearson’s coefficient = 0.53 ± 0.04) at 72 h PI, with no significant difference. However, the extent of colocalization between *β*III tubulin and F-actin in growth cones following 5% stretch injury was significantly higher at 72 h (Pearson’s coefficient 0.65 ± 0.02) compared to growth cones following 0.5% stretch injury and the control (p<0.05, Fig 5B).

### Repetitive very mild stretch injury exacerbates growth cone collapse

Knowing that the growth cones following mild and very mild stretch injury exhibited different cytoskeletal profile, smaller size and increased proportion of collapsed profiles when compared to the growth cones in the uninjured cultures, we investigated the response of growth cones to repetitive very mild (2×0.5%) stretch injury. Axons that received a repetitive insult were stretched again 24 h after the first stretch injury and evaluated at 72 h post the first injury (Fig 1B). We found that the size of the growth cones following single injury (14.59 ± 2.03 *μ*m^2^) and repetitive injury (16.54 ± 0.93 *μ*m^2^) were both significantly smaller compared to the control (22.40 ± 1.89 *μ*m^2^) (Fig 3). However, there was no significant difference between the size of growth cones following single injury and repetitive injury. We then determined the percentage of collapsed growth cones following repetitive injury and compared these with both single injury and uninjured control. Our results show that the percentage of collapsed growth cones in cultures following repetitive injury (94.10 ± 3.02%) was significantly higher compared to the control, uninjured cultures (32.38 ± 3.72%) (p<0.05, Fig 5A). Most importantly, the percentages of collapsed growth cones in cultures following repetitive injury was significant higher than following a single injury (68.33 ± 6.31%) (p<0.05, Fig 5A). Additionally, there was a significant increase of colocalization of F-actin and *β*III tubulin in growth cones following a repetitive very mild insult (Pearson’s coefficient = 0.67 ± 0.03), as compared with both the single, very mild insult (Pearson’s coefficient = 0.57 ± 0.03) and uninjured control (Pearson’s coefficient = 0.53 ± 0.04) (Fig 5B).

### EpoD significantly reduced axonal fragmentation following localized mild axonal stretch injury

**Fig 6:**
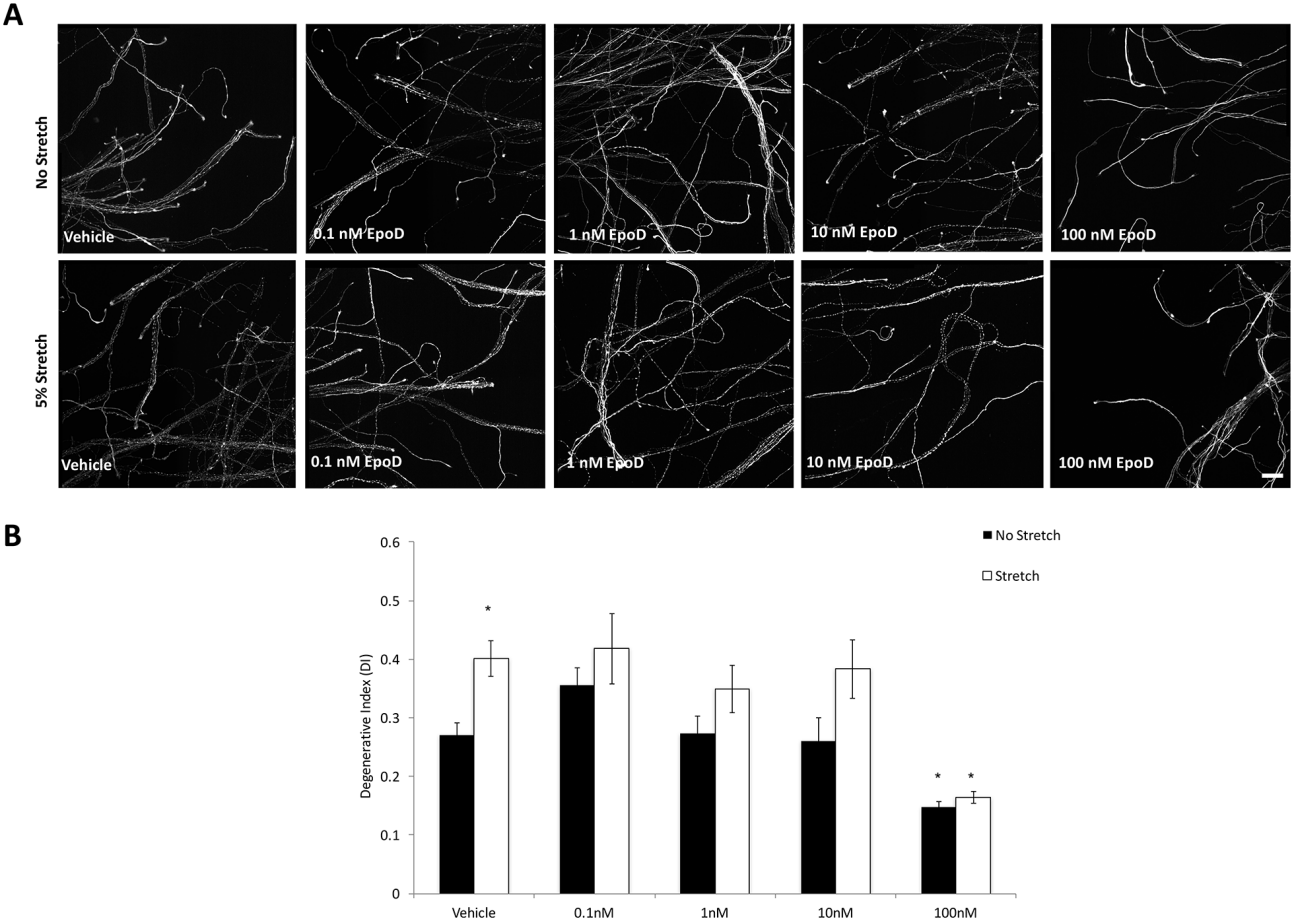
Tau (microtubule marker) immunofluorescence labeling demonstrating the effect of EpoD exposure on 5% stretched axons at 7 DIV. **(A)** Representative fluorescence images of unstretched and stretched culture 24 h after the indicated treatment. **(B)** EpoD (100 nM) applied to axon compartment alone for 24 h induced a significant decrease in degenerative index in the axon compartment when compared to vehicle- treated cultures following injury. The arterisk indicates the difference from paired control. *p<0.05. Error bar = mean ± SEM. Scale bar = 30 *μ*m.

To quantify the effect of the microtubule-stabilizing drug EpoD, on post stretch injury distal axonal responses, we investigated tau immunolabelled images of fixed axons using a particle analyzer algorithm of Image J software. Previous study has indicated that EpoD treatment *in vitro* at concentration range from 0.1 nM to 100 nM do not affect neuron viability, metabolic health or cellular health [17]. Therefore, we investigate the effect of EpoD to axons following stretch injury using concentration within this range (0.1 nM to 100 nM). Axons were first subjected to mild stretch injury (5%) at 7 DIV as described previously. EpoD or vehicle was immediately added to the axonal compartment immediately after stretch injury. Cultures were fixed 24 h later for immunocytochemistry. In injured vehicle-treated cultures we observed signs of degeneration including beading and fragmentation at 24 h after injury. Conversely, the majority of the axons appeared intact in the injured 100 nM EpoD treated cultures (Fig 6A). Quantitative analysis demonstrated 100 nM EpoD substantially influenced the extent of distal axon degeneration. The degenerative index of the 100 nM EpoD-treated injured cultures (DI = 0.16 ± 0.01) was significantly decreased compared to the vehicle-treated injured cultures (DI = 0.40 ± 0.03) (p<0.05, Fig 6B). Similarly, statistical analysis indicated a significant difference between the extent of distal axon degenerative index in the EpoD-treated cultures (DI = 0.15 ± 0.01) and vehicle treated cultures (DI = 0.27 ± 0.02) at 24 h without any stretch injury (p<0.05, Fig 6B). However, we did not observe a significant decrease in the degenerative index in response to other EpoD concentration (0.1, 1 and 10 nM). Taken together, our results indicate that 100 nM EpoD can potentially reduce axon fragmentation in stretch injured cultures.

## Discussion

TBI is an insult to the brain caused by brain deformation, stretching, compression or shear forces as a result of falls, vehicle accidents, sports, assault and gunshot wounds [18]. It is normally characterized into “mild”, “moderate” or “severe” with the majority of TBI cases being categorized as mTBI. In addition, repetitive mTBI or concussion has recently been highlighted as a significant public health problem and has received significant media attention for years particularly with its association with high impact sports [19]. Repetitive concussion has been linked to a distinct neurodegenerative disease known as chronic traumatic encephalopathy (CTE) which can have persistent cognitive, behavioral and pyschiatric effects [20]. For example, a study on soccer players has shown that the number of concussions was inversely related to memory and scores on a visuoperceptual test [21]. In addition, there is experimental evidence to show that repetitive brain injury also increases the susceptibility to chronic TBI as well as other neurodegenerative diseases such as dementia [22] and Alzheimer’s disease [23]. Several *in vivo* and *in vitro* models have therefore been developed to investigate the pathobiological mechanism of TBI and repetitive mTBI at the cellular, subcellular and whole animal levels [24].

*In vitro* experiments are important to achieve a better understanding of the cellular mechanisms that contribute to repetitive concussion related cellular dysfunction. We have recently developed a new *in vitro* model for studying localised axonal stretch injury using a microfluidic device to selectively culture axons on a thin, flexible PDMS membrane, which can be deflected upward to stretch the axons at a range of level [9]. In addition, the fluidic isolation properties of this stretch injury microfluidic device also allow independent drug treatment on axon side and/or soma side, hence providing valuable tool for testing of potential therapeutic agents for TBI. Previous studies show that axon regeneration following injury is driven by the forward movement of the growth cones, a specialized motile structure that located at the tip of growing axons [25–28]. The effects of mild and repetitive mild axonal stretch injury on the growth cones at the tip of axons, however, are currently unclear. Here, we investigate the alterations in growth cone morphology and cytoskeleton profile after single and repetitive stretch injury using our *in vitro* model.

We found that growth cones on the tips of axons following mild and very mild stretch injuries were smaller compared to the growth cones of unstretched axons. This observation is similar to other studies where the growth cones of the tips of regenerative sprouts after axotomy were smaller compared to growth cones of developing axons [29]. In addition, we found that both very mild and mild axonal stretch injury resulted in an increase in the percentage of collapsed growth cones compared to the uninjured control, suggesting that stretch injury even at very mild levels of strain can trigger growth cone collapse.

Differences were not only noticeable in the size and the percentage of collapsed growth cones, but also in their cytoskeletal organization. Here, we found that a mild stretch injury resulted in significantly greater extent of colocalization of actin and microtubules in growth cones and greater proportion of collapsed growth cones compared to the axons that had received very mild injuries at 72 h PI. The extent of colocalization of actin and microtubules in growth cones of the axons changes following mild injury suggesting that stretch injury change cytoskeletal actin and microtubule dynamics. Therefore, following mild stretch injury, the majority of the growth cones formed were dystrophic or collapsed, with little microtubule extension into the periphery region of growth cones, and retraction of filopodia. These collapsed growth cones are also known as “retraction bulbs” that lack the actin rich filopodia and lamellipodia and hence loose the ability to detect guidance cues [16, 30]. Retraction bulbs are considered important hallmarks of failure to regenerate in TBI, as well as in other neurodegenerative diseases such as multiple sclerosis, Alzheimer’s disease and Parkinson’s disease [16, 31, 32]. These bulbs are typically round or oval shaped and lack any kind of extensions. We found that both very mild and mild axonal stretch injury resulted in an increase in the percentage of collapsed growth cones compared to the control at both 24 h and 72 h PI, suggesting that stretch injury even at very mild levels of strain can trigger the formation of retraction bulb. Previous studies show that mild stretching induces damage to microtubules and as such causes failure of axonal transport and leads to axon degeneration [33, 34]. Therefore, we suggest that in the current study, mild axonal stretch injury had higher number of retraction bulbs, possibly due to the damage to the microtubules, leaving the cell membrane unsupported.

On the other hand, growth cones on the tips of axons that have received very mild stretch injury have similar localization of microtubule and F-actin at both 24 h and 72 PI as growth cones on uninjured, developing axons, indicated by phalloidin staining and *β*III tubulin labelling. This suggests that growth cones formed following very mild axonal stretch injury possess the cytoskeletal capacity for motility and extension similar to normal developing growth cones [35]. A previous study using our model [9] also observed dendritic beading along the dendrite shaft and irregular microtubule associated protein 2 (MAP 2) expression in the soma compartment following very mild injury (0.5%) at 24 h PI stretched neurons. Dendritic alterations have also been observed in previous studies performing *in vitro* axonal stretch injury by the Smith’s group [36] and in animal models of TBI [37–39]. Therefore, we suggest that distal axonal stretch injury triggers injury to both distal and proximal part of neurons. However, there is previous studies show that axon degeneration has a degree of independence from the cell body and importantly degeneration of the axon can occur without apoptosis [40, 41]. Thus, a better understanding of the mechanism of axon degeneration and protection in addition to neuronal cell body protection and combinatorial approaches may be necessary to design successful therapeutic strategies for TBI.

A number of clinical and experimental investigations have described the behaviour, physiological, pathological sequelae of mild repetitive head injury on macroscopic and microscopic levels [2, 6, 19]. Recently, in a study by Shitaka *et al*. [42] using an *in vivo* controlled cortical impact model, two mild injuries were administered to mice 24 h apart and significant increased cognitive deficits were demonstrated after repeated injuries. Another repetitive injury study using an *in vitro* axonal stretch injury model also observed significant increased intracellular calcium that led to degeneration of axons after the application of a second, identical mild stretch injury (3%) 24 h following an initial mild injury [8]. Here, we used a similar double insult timelime by stretching the cells 24 h after their initial insult. After a single, very mild injury (0.5%), we found that the extent of colocalization of *β*III tubulin and F-actin in growth cones was similar to the growth cones in normal developing distal axons. When after 24 h a second, very mild injury is applied, both the extent of colocalization of *β*III tubulin and F-actin in growth cones and the proportion of collapsed growth cones significantly increased at 72 h PI when compared with the control. These results demonstrate that even at a very mild strain level the growth cone response in repetitive very mild stretch injury was greatly increased compared to single injury. Previous experiments demonstrated that the animals that receive repeated injuries seven days apart do not exhibit cognitive deficit, suggesting that the brain can recover from first injury if given sufficient amount of time [43]. *In vivo* investigations by Povlishock and colleagues [44] also show that axons exhibited axonal swelling at 7 days following moderate fluid percussion injury in the adult cat, but significantly reduced at 14 days post injury and had significant increased growth associated protein (GAP43) immunoreactivity (a regenerative response marker). Our results suggest that 24 h is not sufficient for the axons to recover and re-establish cytoskeletal structures and processes such as axon transport or calcium signalling even though the injury was very mild. Future investigation therefore may require a longer time period to investigate the recovery mechanism of axons following very mild axonal stretch injury. For example, repeated injuries studies can be applied at three days or more after the first injury.

It is well known that cytoskeletal elements play an important role in maintaining neuronal function. Hence, it is not surprising that following TBI, a number a number of cytoskeletal changes may be present including abnormal accumulations of cytoskeletal proteins within the axon, abnormal phosphorylation and mislocalization of proteins within the soma [45–46]. Previous studies also show the loss of microtubule associated proteins such as Tau and MAP2 are associated with TBI [1, 25, 47, 48]. Results from the current study also show that axonal stretch injury induced microtubule defects that lead to the formation of axonal bulb structures which is similar to those observed previously using the *in vitro* model developed by Smith group [7]. The alterations in microtubule in TBI suggests that after injury, treatment with compounds that stabilize microtubules can potentially modify a range of different aspects of brain’s response to trauma. Previous studies using Taxol have demonstrated the protective role of microtubule stabilizing drugs in the prevention of the formation of axon retraction bulbs after spinal cord injury [16]. In addition, King and colleague have shown that microtubule stabilization of axons using Taxol resulted in a significant reduction in the number of fragmented axons following excitotoxicity [49]. Adlard and colleagues [50] also demonstrated that taxol inhibits microtubule loss in rodent experimental model. Therefore, in this study, we investigated the potential use of EpoD in treatment of stretch injury related trauma. Unlike Taxol, this microtubule-stabilizing drug has been suggested to cross the blood brain barrier and is retained within the CNS for several days as evidenced by its use in a mouse model of schizophrenia [51, 52]. In addition, Brunden and colleagues [52] also demonstrate that administration of EpoD to a transgenic mouse model of tauopathy significantly improve microtubule density and axonal integrity and cognitive performance without inducing notable side effect. A previous study has also demonstrated the protective role of microtubule stabilization with EpoD on cortical neurons following axonal transection *in vitro* model through significantly increased number of axonal sprouts [17]. However, the role of microtubules stabilization specifically in the axon compartment was not examined. Here, our study shows that high concentration of EpoD (100 nM) treatment for 24 h to the axon compartment following mild axonal stretch injury significantly decreases microtubule fragmentation compared to vehicle treated cultures. Conversely, the administration of lower concentration of EpoD (0.1 nM, 1 nM and 10 nM) had no significant effect on the axon integrity. Previously, Brizuela and coworkers [17] demonstrated that EpoD (100 nM) in uninjured cortical neurons *in vitro* resulted in a significant initial increase in acetylated tubulin (marker of stable polymerized microtubules) and loss of Tau (microtubule associated protein marker) by 24 h treatment. Therefore, our data suggests that 100 nM EpoD may promote both the polymerization and stabilization of microtubule following stretch injury and hence reduce the distal degenerative response. Interestingly the formation of retraction bulbs at the tips of injured axons or axon bulbs (axon swelling) in an injured axon has been associated with disruption of microtubules [16, 33]. Therefore, future investigations are required to investigate the effect of EpoD treatment on the formation of axon swelling. On the other hand, Jang and colleagues [53] demonstrated that 0.1 nM of EpoB to embryonic cortical neurons resulted in higher neuronal viability and promoted axon growth comparing to neurons with 100 nM EpoB treatment at DIV 3. Therefore, future experiments aimed at testing both the brain barrier penetrating drugs, EpoB and EpoD, in both the axon and soma compartments, at differing concentrations, would be of value.

In summary, experiments in this study were conducted using a novel model of axonal stretch injury that is highly adaptable and has the ability to apply both chemical and physical insult to both soma compartment and axon compartments in a highly targeted manner. Our results suggest that growth cones at the tips of axons exhibit a different cytoskeletal profile and characteristics following very mild and mild axonal stretch injury. Particularly following mild or repetitive very mild axonal stretch injury, the tip of distal axons formed abnormal and dysfunctional retraction bulb. These alterations were similar to those observed *in vivo* and confirmed the suitability of this system for studying the neuronal responses to discrete axonal stretch injury. Unfortunately, the optical transparency properties of our device are not compatible with high resolution live time lapse analysis. Therefore, it is difficult to distinguish the effects that occur in post-injury axonal tips/growth cones from those that are occurring within pre-existing structures. Furthermore, the current device does not allow the investigation of synaptically-connected axons from two different neuronal populations, as you would expect *in vivo*. ‘Second generation’ devices, will be explicitly designed to overcome these limitations. Our investigations also indicate that EpoD, a brain penetrant drug that has immediate appropriate clinical applications may find therapeutic efficacy in TBI. However, further investigations using *in vivo* animal models of injury are necessary. In conclusion, this model is amenable to revealing further insights into the cellular changes triggered by axonal injury. However, this model has the limitation that stretch injury was applied to axons without post synaptic partners. Further studies will integrate a diode fabrication approach [54], which will enable us to integrate two distinct neuron populations that will form functional synapses within the chamber and hence allow us to apply stretch injury to synaptically-connected axons and will provide further insights into the changes occurring post injury. Furthermore, future investigations applying different levels of strain, altering the age and anatomical sources of the neurons and the position of the pneumatic valve relative to the soma, as well as co-culturing with glial cells will also likely contribute to a much greater understanding of the complex cascade of cellular mechanisms underpinning the response to TBI. Such a model also has a great potential for the discovery of axon or soma protective therapies following CNS injury.

## Supporting information

S1 Fig Quantification method of growth cone area. **(A)** The phalloidin stained growth cone image was opened in image J in grayscale. **(B)** The image was then thresholded using automated command: process>binary>make binary. **(C)** Freehand line module was used to draw the outline of the growth cone through the binary image and then use the command: analyze>measure was used to measure the area. Scale was set prior to analysis.
(TIF)

## References

[1] Smith DH, Hicks R, Povlishock JT. Therapy development for diffuse axonal injury. Journal of Neurotrauma. 2013;30(5):307–23. 10.1089/neu.2012.2825 23252624 PMC3627407

[2] Huh JW, Widing AG, Raghupath R. Repetitive mild non-contusive brain trauma in immature rats exacerbates traumatic axonal injury and axonal calpain activation: A preliminary report. J Neurotrauma. 2007;24(1):15–27. 10.1089/neu.2006.0072 17263667

[3] Laurer HL, Bareyre FM, Lee V, Trojanowski JQ, Longhi L, Hoover R, et al Mild head injury increasing the brain's vulnerability to a second concussive impact. J Neurosurg. 2001;95(5):859–70. 10.3171/jns.2001.95.5.0859 11702878

[4] DeFord SM, Wilson MS, Rice AC, Clausen T, Rice LK, Barabnova A, et al Repeated mild brain injuries result in cognitive impairment in B6C3F1 mice. J Neurotrauma. 2002;19(4):427–38. 10.1089/08977150252932389 11990349

[5] Ellis EF, McKinney JS, Willoughby KA, Liang S, Povlishock JT. A new model for rapid stretch-induced injury of cells in culture-characterization of the model using astrocytes. J Neurotrauma. 1995;12(3):325–39. 10.1089/neu.1995.12.325 7473807

[6] Slemmer JE, Matser EJT, De Zeeuw CI, Weber JT. Repeated mild injury causes cumulative damage to hippocampal cells. Brain. 2002;125:2699–709. 12429597 10.1093/brain/awf271

[7] Smith DH, Wolf JA, Lusardi TA, Lee VMY, Meaney DF. High tolerance and delayed elastic response of cultured axons to dynamic stretch injury. J Neurosci. 1999;19(11):4263–9. 10341230 10.1523/JNEUROSCI.19-11-04263.1999 PMC6782601

[8] Yuen TJ, Browne KD, Iwata A, Smith DH. Sodium channelopathy induced by mild axonal trauma worsens outcome after a repeat Injury. J Neurosci Res. 2009;87(16):3620–5. 10.1002/jnr.22161 19565655 PMC3014254

[9] Yap YC, Dickson TC, King AE, Breadmore MC, Guijt RM. Microfluidic culture platform for studying neuronal response to mild to very mild axonal stretch injury. Biomicrofluidics. 2014;8:044110 10.1063/1.4891098 25379095 PMC4189213

[10] Taylor AM, Rhee SW, Tu CH, Cribbs DH, Cotman CW, Jeon NL. Microfluidic multicompartment device for neuroscience research. Langmuir. 2003;19(5):1551–6. 10.1021/la026417v 20725530 PMC2923462

[11] Unger MA, Chou HP, Thorsen T, Scherer A, Quake SR. Monolithic microfabricated valves and pumps by multilayer soft lithography. Science. 2000;288(5463):113–6. 10753110 10.1126/science.288.5463.113

[12] Taylor AM, Blurton-Jones M, Rhee SW, Cribbs DH, Cotman CW, Jeon NL. A microfluidic culture platform for CNS axonal injury, regeneration and transport. Nat Methods. 2005;2(8):599–605. 10.1038/nmeth777 16094385 PMC1558906

[13] Richter M, Murai KK, Bourgin C, Pak DT, Pasquale EB. The EphA4 receptor regulates neuronal morphology through SPAR-mediated inactivation of Rap GTPases. J Neurosci. 2007;27(51):14205–15. 10.1523/JNEUROSCI.2746-07.2007 18094260 PMC6673515

[14] Sasaki Y, Vohra BPS, Lund FE, Milbrandt J. Nicotinamide mononucleotide adenylyl transferase-mediated axonal protection requires enzymatic activity but not increased levels of neuronal nicotinamide adenine dinucleotide. J Neurosci. 2009;29(17):5525–35. 10.1523/JNEUROSCI.5469-08.2009 19403820 PMC3162248

[15] Goedert M, Crowther RA, Garner CC. Molecular characterization of microtubule-associated proteins TAU and MAP2. Trends Neurosci. 1991;14(5):193–9. 1713721 10.1016/0166-2236(91)90105-4

[16] Erturk A, Hellal F, Enes J, Bradke F. Disorganized microtubules underlie the formation of retraction bulbs and the failure of axonal regeneration. J Neurosci. 2007;27(34):9169–80. 10.1523/JNEUROSCI.0612-07.2007 17715353 PMC6672197

[17] Brizuela M, Blizzard CA, Chuckowree JA, Dawkins E, Gasperini RJ, Young KM, et al The microtubule-stabilizing drug Epothilone D increases axonal sprouting following transection injury in vitro. Molecular and Cellular Neuroscience. 2015;66:129–40. 10.1016/j.mcn.2015.02.006 25684676

[18] Johnson VE, Stewart W, Smith DH. Axonal pathology in traumatic brain injury. Exp Neurol. 2013;246:35–43. 10.1016/j.expneurol.2012.01.013 22285252 PMC3979341

[19] Mannix R, Meehan WP, Mandeville J, Grant PE, Gray T, Berglass J, et al Clinical correlates in an experimental model of repetitive mild brain Injury. Ann Neurol. 2013;74(1):65–75. 10.1002/ana.23858 23922306 PMC6312716

[20] McKee AC, Cantu RC, Nowinski CJ, Hedley-Whyte ET, Gavett BE, Budson AE, et al Chronic traumatic encephalopathy in athletes: progressive tauopathy after repetitive head injury. J Neuropathol Exp Neurol. 2009;68(7):709–35. 10.1097/NEN.0b013e3181a9d503 19535999 PMC2945234

[21] Matser JT, Kessels AGH, Jordan BD, Lezak MD, Troost J. Chronic traumatic brain injury in professional soccer players. Neurology. 1998;51(3):791–6. 9748028 10.1212/wnl.51.3.791

[22] Jordan BD. Chronic traumatic brain injury associated with boxing. Semin Neurol. 2000;20(2):179–85. 10.1055/s-2000-9826 10946737

[23] Uryu K, Laurer H, McIntosh T, Pratico D, Martinez D, Leight S, et al Repetitive mild brain trauma accelerates A beta deposition, lipid peroxidation, and cognitive impairment in a transgenic mouse model of Alzheimer amyloidosis. J Neurosci. 2002;22(2):446–54. 11784789 10.1523/JNEUROSCI.22-02-00446.2002 PMC6758680

[24] Weber JT. Experimental models of repetitive brain injuries. Neurotrauma: New Insights into Pathology and Treatment. 2007;161:253–61. 10.1016/S0079-6123(06)61018-2 17618983

[25] Bradke F, Fawcett JW, Spira ME. Assembly of a new growth cone after axotomy: the precursor to axon regeneration. Nature Reviews Neuroscience. 2012;13(3):183–93. 10.1038/nrn3176 22334213

[26] Dent EW, Gertler FB. Cytoskeletal dynamics and transport in growth cone motility and axon guidance. Neuron. 2003;40(2):209–27. 14556705 10.1016/s0896-6273(03)00633-0

[27] Gordon-Weeks PR. Microtubules and growth cone function. J Neurobiol. 2004;58(1):70–83. 10.1002/neu.10266 14598371

[28] Vickers JC, King AE, Woodhouse A, Kirkcaldie MT, Staal JA, McCormack GH, et al Axonopathy and cytoskeletal disruption in degenerative diseases of the central nervous system. Brain Research Bulletin. 2009;80(4–5):217–23. 10.1016/j.brainresbull.2009.08.004 19683034

[29] Blizzard CA, Haas MA, Vickers JC, Dickson TC. Cellular dynamics underlying regeneration of damaged axons differs from initial axon development. Eur J Neurosci. 2007;26(5):1100–8. 10.1111/j.1460-9568.2007.05750.x 17767489

[30] Hill CE, Beattie MS, Bresnahan JC. Degeneration and sprouting of identified descending supraspinal axons after contusive spinal cord injury in the rat. Exp Neurol. 2001;171(1):153–69. 10.1006/exnr.2001.7734 11520130

[31] Coleman M. Axon degeneration mechanisms: Commonality amid diversity. Nature Reviews Neuroscience. 2005;6(11):889–98. 10.1038/nrn1788 16224497

[32] Mueller BK, Mueller R, Schoemaker H. Stimulating neuroregeneration as a therapeutic drug approach for traumatic brain injury. Br J Pharmacol. 2009;157(5):675–85. 10.1111/j.1476-5381.2009.00220.x 19422372 PMC2721253

[33] Tang-Schomer MD, Patel AR, Baas PW, Smith DH. Mechanical breaking of microtubules in axons during dynamic stretch injury underlies delayed elasticity, microtubule disassembly, and axon degeneration. FASEB J. 2010;24(5):1401–10. 10.1096/fj.09-142844 20019243 PMC2879950

[34] Tang-Schomer MD, Johnson VE, Baas PW, Stewart W, Smith DH. Partial interruption of axonal transport due to microtubule breakage accounts for the formation of periodic varicosities after traumatic axonal injury. Exp Neurol. 2012;233(1):364–72. 10.1016/j.expneurol.2011.10.030 22079153 PMC3979336

[35] Chuckowree JA, Vickers JC. Cytoskeletal and morphological alterations underlying axonal sprouting after localized transection of cortical neuron axons in vitro. J Neurosci. 2003;23(9):3715–25. 12736342 10.1523/JNEUROSCI.23-09-03715.2003 PMC6742187

[36] Monnerie H, Tang-Schomer MD, Iwata A, Smith DH, Kim HA, Le Roux PD. Dendritic alterations after dynamic axonal stretch injury in vitro. Exp Neurol. 2010;224(2):415–23. 10.1016/j.expneurol.2010.05.001 20478308 PMC3979358

[37] Saatman KE, Graham DI, McIntosh TK. The neuronal cytoskeleton is at risk after mild and moderate brain injury. J Neurotrauma. 1998;15(12):1047–58. 10.1089/neu.1998.15.1047 9872461

[38] Posmantur R, Hayes RL, Dixon CE, Taft WC. Neurofilament-68 and neurofilament-200 protein levels decrease after traumatic brain injury. J Neurotrauma. 1994;11(5):533–45. 10.1089/neu.1994.11.533 7861446

[39] Folkerts MM, Berman RF, Muizelaar JP, Rafols JA. Disruption of MAP-2 immunostaining in rat hippocampus after traumatic brain injury. J Neurotrauma. 1998;15(5):349–63. 10.1089/neu.1998.15.349 9605349

[40] Osterloh JM, Yang J, Rooney TM, Fox AN, Adalbert R, Powell EH, et al dSarm/Sarm1 Is Required for Activation of an Injury-Induced Axon Death Pathway. Science. 2012;337(6093):481–4. 10.1126/science.1223899 22678360 PMC5225956

[41] Finn JT, Weil M, Archer F, Siman R, Srinivasan A, Raff MC. Evidence that wallerian degeneration and localized axon degeneration induced by local neurotrophin deprivation do not involve caspases. Journal of Neuroscience. 2000;20(4):1333–41. 10662823 10.1523/JNEUROSCI.20-04-01333.2000 PMC6772375

[42] Shitaka Y, Tran HT, Bennett RE, Sanchez L, Levy MA, Dikranian K, et al Repetitive closed-skull traumatic brain injury in mice causes persistent multifocal axonal injury and microglial reactivity. J Neuropathol Exp Neurol. 2011;70(7):551–67. 10.1097/NEN.0b013e31821f891f 21666502 PMC3118973

[43] Longhi L, Saatman KE, Fujimoto S, Raghupathi R, Meaney DF, Davis J, et al Temporal window of vulnerability to repetitive experimental concussive brain injury. Neurosurgery. 2005;56(2):364–73. 15670384 10.1227/01.neu.0000149008.73513.44

[44] Christman CW, Salvant JB, Walker SA, Povlishock JT. Characterization of a prolonged regenerative attempt by diffusely injured axons following traumatic brain injury in adult cat: a light and electron microscopic immunocytochemical study. Acta Neuropathologica. 1997;94(4):329–37. 9341933 10.1007/s004010050715

[45] Miller CCJ, Ackerley S, Brownlees J, Grierson AJ, Jacobsen NJO, Thornhill P. Axonal transport of neurofilaments in normal and disease states. Cellular and Molecular Life Sciences. 2002;59(2):323–30. 11924605 10.1007/s00018-002-8425-7 PMC11146161

[46] Maxwell WL, Graham DI. Loss of axonal microtubules and neurofilaments after stretch-injury to Guinea pig optic nerve fibers. Journal of Neurotrauma. 1997;14(9):603–14. 10.1089/neu.1997.14.603 9337123

[47] King CE, Adlard PA, Dickson TC, Vickers JC. Neuronal response to physical injury and its relationship to the pathology of Alzheimer's disease. Clinical and Experimental Pharmacology and Physiology. 2000;27(7):548–52. 10874516 10.1046/j.1440-1681.2000.03292.x

[48] Farkas O, Povlishock JT. Cellular and subcellular change evoked by diffuse traumatic brain injury: a complex web of change extending far beyond focal damage. In: Weber JT, Maas AIR, editors. Neurotrauma: New Insights into Pathology and Treatment. Progress in Brain Research. 1612007. p. 43–59. 10.1016/S0079-6123(06)61004-2 17618969

[49] Childs WR, Motala MJ, Lee KJ, Nuzzo RG. Masterless soft lithography: Patterning UV/ozone-induced adhesion on poly(dimethylsiloxane) surfaces. Langmuir. 2005;21(22):10096–105. 10.1021/la050011b 16229532

[50] Adlard PA, King CE, Vickers JC. The effects of taxol on the central nervous system response to physical injury. Acta Neuropathologica. 2000;100(2):183–8. 10963366 10.1007/s004019900160

[51] Andrieux A, Salin P, Schweitzer A, Begou M, Pachoud B, Brun P, et al Microtubule stabilizer ameliorates synaptic function and behavior in a mouse model for schizophrenia. Biological Psychiatry. 2006;60(11):1224–30. 10.1016/j.biopsych.2006.03.048 16806091

[52] Brunden KR, Zhang B, Carroll J, Yao YM, Potuzak JS, Hogan AML, et al Epothilone D Improves Microtubule Density, Axonal Integrity, and Cognition in a Transgenic Mouse Model of Tauopathy. Journal of Neuroscience. 2010;30(41):13861–6. 10.1523/JNEUROSCI.3059-10.2010 20943926 PMC2958430

[53] Jang EH, Sim A, Im SK, Hur EM. Effects of Microtubule Stabilization by Epothilone B Depend on the Type and Age of Neurons. Neural Plasticity. 2016. 10.1155/2016/5056418 PMC5107872 27872763

[54] Peyrin JM, Deleglise B, Saias L, Vignes M, Gougis P, Magnifico S, et al Axon diodes for the reconstruction of oriented neuronal networks in microfluidic chambers. Lab on a Chip. 2011;11(21):3663–73. 10.1039/c1lc20014c 21922081

