# Supplementary figures and images for: Mild and repetitive very mild axonal stretch injury triggers cystoskeletal mislocalization and growth cone collapse
Source: PLoS One. 2017 May 4;12(5):e0176997. doi: 10.1371/journal.pone.0176997 (PMC5417565; doi:10.1371/journal.pone.0176997)

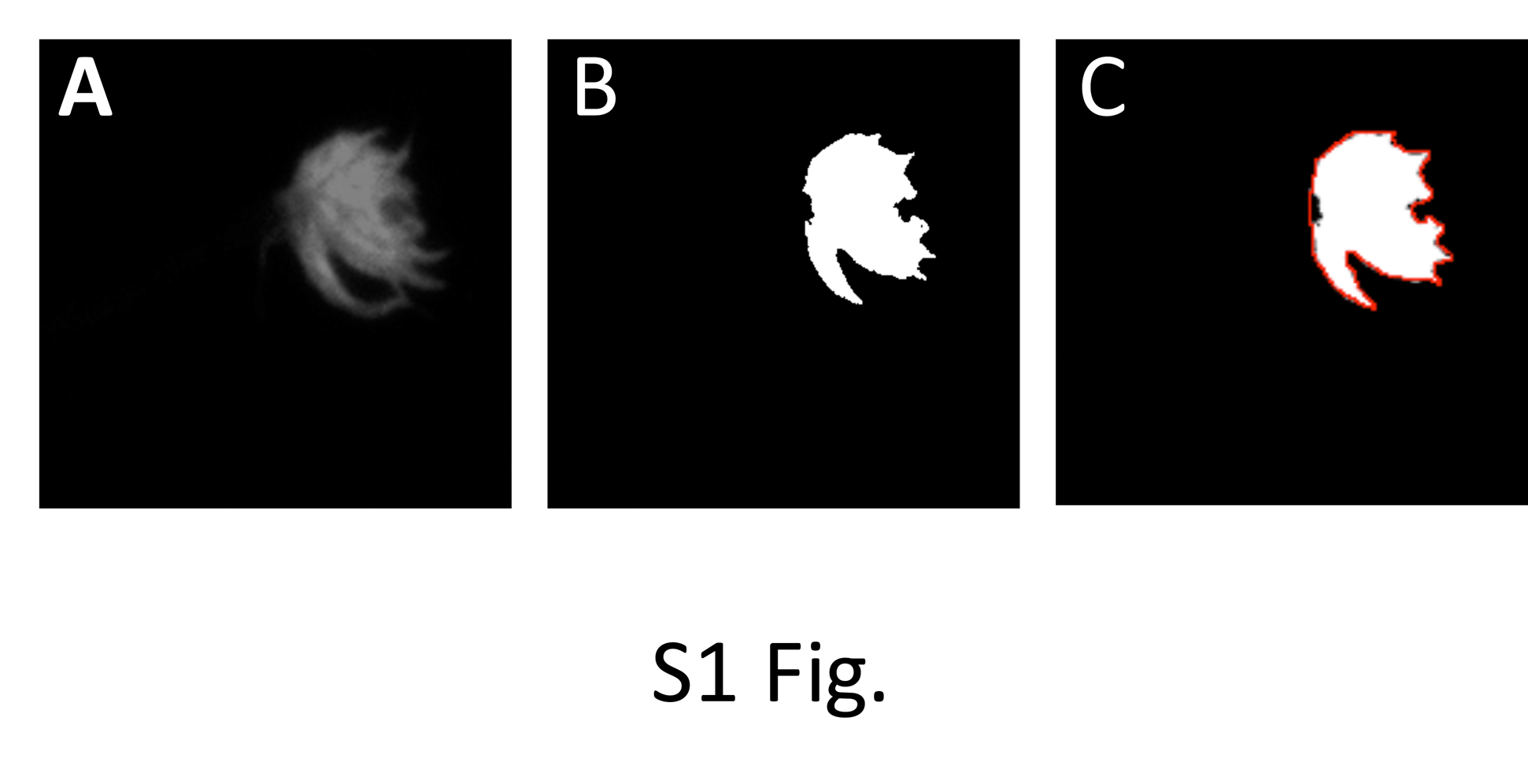

Supplement: S1 Fig — (A) The phalloidin stained growth cone image was opened in image J in grayscale. (B) The image was then thresholded using automated command: process>binary>make binary. (C) Freehand line module was used to draw the outline of the growth cone through the binary image and then use the command: analyze>measure was used to measure the area. Scale was set prior to analysis. (TIF) [file pone.0176997.s001.tif]
